# Collagen triple helix repeat containing 1 (CTHRC1) activates Integrin β3/FAK signaling and promotes metastasis in ovarian cancer

**DOI:** 10.1186/s13048-017-0358-8

**Published:** 2017-10-11

**Authors:** Biying Guo, Huan Yan, Luying Li, Kemin Yin, Fang Ji, Shu Zhang

**Affiliations:** 1 Department of Gynecology and Obstetrics, Shanghai Key Laboratory of Gynecology Oncology, Renji Hospital, School of Medicine, Shanghai Jiao Tong University, PuJian Road No.160, Shanghai, 200127 China; 2 Department of Gynecology and Obstetrics, Shanghai First Maternity and Infant Hospital, School of Medicine, Shanghai Tong Ji University, Shanghai, 201204 China

**Keywords:** CTHRC1, Ovarian cancer, Metastasis, Integrin/FAK signaling

## Abstract

**Background:**

Metastasis is the major cause of morbidity and mortality in patients with epithelial ovarian cancer (EOC), however the mechanisms that underline this process are poorly understood. Collagen triple helix repeat containing-1 (CTHRC1) is a 28-kDa secreted protein reported to be involved in vascular remodeling, bone formation and morphogenesis. This study aimed to investigate the role of CTHRC1 in promoting the metastasis of EOC and to elucidate the underlying molecular mechanisms.

**Methods:**

The biologic functions of CTHRC1 in metastasis were validated both in vivo and in vitro experiments. The phosphor-antibody microarray analysis and Co-immunoprecipitation were performed to detect and identify the integrin β3/FAK signaling pathway that mediated the function of CTHRC1. Seventy two EOC samples were analyzed for association between CTHRC1/integrin β3 expression and patient clinicopathological features.

**Results:**

We demonstrated that CTHRC1 enhances the biological behavior of EOC including cell migration, invasion, as well as its adhesion capability to cell-extracellular matrix in vitro. Additionally, CTHRC1 promoted metastatic spread of EOC cells in an i.p. ovarian xenograft model and this phenotype was primarily ascribed to the activation of integrin/FAK signaling. Mechanistically, we determined that FAK were phosphorylated on Tyr397, and were activated by integrin β3, which is important for the CTHRC1-mediated migratory and invasive ability of EOC cells in vitro and i.p. metastasis. In addition, we found that attenuated CTHRC1/integrin β3 expression predicted a poor prognostic phenotype and advanced clinical stage of EOC.

**Conclusions:**

Our results suggest that CTHRC1, a newly identified regulator of i.p. metastasis through activation of integrin β3/FAK signaling in EOC, may represent a potential therapeutic target for ovarian cancer.

**Electronic supplementary material:**

The online version of this article (10.1186/s13048-017-0358-8) contains supplementary material, which is available to authorized users.

## Background

Ovarian cancer accounts for about 3% of all cancers among women, and it is the most deadly gynecologic cancer in female population worldwide [1]. The most common type is epithelial ovarian cancer (EOC). Staging in EOC begins with Stage I, and gradually progresses in severity to stage IV, which is the end-stage, and entails the spread outside of the abdomen. Seventy five percent of patients present at an advanced (Stage III or IV) with metastasis commonly observed within the peritoneal cavity that leads to variety of conditions including ascites and small bowel obstruction [2, 3]. Although many efforts have been made to treat peritoneal dissemination of ovarian cancer, such as debulking surgery and systemic or intraperitoneal chemotherapy, effective eradication of peritoneal dissemination remains a major challenge in the clinical management of ovarian cancer. Metastasis is the major cause of morbidity and mortality in patients with EOC, however the mechanisms that underline this process in EOC are poorly understood.

Cancer metastasis is a key step in cancer progression, and it can be divided into two major steps. First step refers to physical translocation of a cancer cell to a distant organ, while the second step includes the process of the development of the cancer cells into a metastatic lesion at the distant site [4, 5]. In EOC, peritoneal metastasis requires modifications of tumor cells to facilitate interaction with the peritoneal stroma and mesothelium. The success of this metastatic step depends on alterations in cell-cell and cell-excretal cellular matrix (ECM) adhesion, epithelial-mesenchymal transition (EMT) and anoikis resistance [4, 6–8]. The cross-talk signaling events between ovarian cancer cells and peritoneum include increased expression of integrins, chemokine receptors (CXCRs), CXC chemokine ligands (CXCLs), matrix metalloproteinases (MMPs), urokinase-type plasminogen activator (uPA) and lysophosphatidic acid [9–12]. Integrins are well-known adhesion molecules, and a large family of heterodimeric transmembrane glycoprotein that has the ability to link the ECM to the intracellular actin cytoskeleton. While binding to multiple compounds of the ECM, integrin recruits downstream targets including the focal adhesion kinase (FAK) [13]. The phosphorylated FAK activates a variety of signaling to mediate cell attachment, survival, motility, proliferation, and invasion [14, 15]. Recently, several studies showed that integrins, particularly integrin β3 receptor, were implicated in the metastasis and invasion of various tumors. Additionally, it was proved that the inhibition of integrin/FAK signaling activation could decrease the migration and invasion of cancer cells [16–18].

Collagen triple helix repeat containing-1 (CTHRC1) is a 28-kDa secreted protein, reported to be involved in vascular remodeling, bone formation and morphogenesis [19]. In addition to functioning in the context of arterial injury, recent studies have reported CTHRC1 acts as a prognostic factor. Furthermore, it promotes tumor progression, migration and adhesion in many human aggressive tumors including pancreatic ductal adenocarcinomas (PDAC), hepatocellular carcinoma (HCC), colorectal cancer, non-small cell lung cancer and ovarian cancer [20–23]. Although CTHRC1 expression has been observed in human solid cancers, the molecular mechanisms underlying CTHRC1 actions in cancer cells is still not entirely clear. Hou et al. and Ma et al. reported that CTHRC1 might activate Wnt signaling to promote metastasis of ovarian cancer and gastrointestinal stromal tumor [23, 24]. Park et al. suggested that CTHRC1 act as an important positive regulator of Src-FAK signaling in pancreatic cancer [25]. It was reported that CTHRC1 promotes invasion capability of colorectal cancer cells via extracellular signal-regulated kinase (ERK)-dependent induction of MMP9 expression [26]. In EOC, we found that CTHRC1 expression is correlated with clinical stage, peritoneal metastasis status and lymph node metastasis, which was consisted with findings reported by Hou et al. [23]. The peritoneal metastasis is uniquely characteristic of EOC, nevertheless the underlying mechanisms have not been properly illustrated. The goal of this study was to analyze the role of CTHRC1 as a mediator of ovarian tumor dissemination in the peritoneal space.

In the following paper we showed for the first time that CTHRC1 enhances the migration and invasive capabilities of EOC cell, and its adhesion to vitronectin by up-regulating integrin β3 and stimulating the FAK phosphorylation. In addition, over-expression of CTHRC1 promotes metastatic spread of EOC cells to the peritoneal surface and mesentery in an i.p. ovarian xenograft model. Also, the inhibition of FAK could suppress the effect of CTHRC1 on i.p tumor seeding in vivo. Furthermore, in parallel with over-expression of integrin β3, CTHRC1 was significantly up-regulated in ovarian cancer tissue, and positively correlated with the FIGO stage, peritoneal metastasis status and lymph node metastasis. These data reveal a novel role for CTHRC1, regulator of i.p. metastasis through activation of integrin β3/FAK signaling in ovarian cancer, as a potential therapeutic target for the disease.

## Methods

### Cell lines and human tissue specimens

The human epithelial ovarian cancer cell lines SKOV3 and ES2 were commercially purchased from the American Type Culture Collection (Rockville, MD, USA). SKOV3 has been established from an ovarian adenocarcinoma and was derived from the ascites of a 64-year-old Caucasian female. ES2 has been isolated from a poorly differentiated ovarian clear-cell carcinoma. A2780 and HO8910 were obtained from the Cell Bank of the Chinese Academy of Sciences (Shanghai, China). A2780 has been established from adenocarcinomas of the ovary and it is the parent line to the cisplatin resistant study in ovarian cancer. HO8910 was derived from the ascites of Chinese patients with ovarian serous adenocarcinomas. IOSE, an immortalized ovarian surface superficial epithelium cell line, was a kind gift from Prof. MW Chan (National Chung Cheng University, Taiwan). Cell lines (A2780, ES2) were cultured in RPMI-1640 and cell lines (SKOV3, HO8910 and IOSE) were cultured in DMEM/High Glucose supplemented with 10% fetal bovine serum (FBS, Gibco) and 1% penicillin-streptomycin (Gibco) at 37 °Cin humidified atmosphere containing 5% CO_2_.

A total of 72 primary epithelial ovarian cancer (PEOC) tissues were collected from patients who underwent surgery at department of Obstetrics and Gynecology, Ren Ji Hospital, Shanghai Jiao Tong University School of Medicine, Shanghai, China. All specimens were collected ahead of chemotherapy, frozen at −80 °C within 1 h after surgery and classified by pathologist to ensure >85% presence of tumor cells. Among the PEOC tissue specimens, 34 specimens were confirmed to be stage I-II, and 38 specimens were confirmed to be stage III-IV. Ten normal ovarian tissues were obtained from patients that had undergone a total hysterectomy with prophylactic oophorectomy. Written informed consent was obtained from each patient, and the use of clinical specimens was approved by the Institutional Ethics Committee.

### Stable transfection

Lenti-CTHRC1 and Lenti-shCTHRC1 were purchased from Genechem (Shanghai, China). SKOV3 cells and HO8910 cells were infected with virus supernatant fluid in complete medium with 5μg/ml polybrene. Stable transfected cells were selected in puromycin for 1 week and verified by Western blot.

### Transwell migration and invasion assays

To verify the cell motility in vitro, 24-well plates and matching upper chambers (Corning, 8μm, USA) were used. As for invasion assays, the chambers were coated with Matrigel (BD Biosciences, USA), then 8×10^4^ SKOV3-CTHRC1 or SKOV3-shCTHRC1 cells or 1×10^5^ HO8910-CTHRC1 cells in serum-free medium were plated in each chamber. As for migration assays, 2×10^4^ cells/chamber of SKOV3-CTHRC1 or SKOV3-shCTHRC1 or 3×10^4^ cells/chamber of HO8910-CTHRC1 in serum-free medium were seeded. 600μl completed medium was added into the lower chamber. After incubating at 37 °C for 24 h, cells in superstratum were wiped and cells on the undersurface were fixed by paraformaldehyde, stained by 0.1% crystal violet and counted in five random fields at 400× magnification. Cell migration and invasion assays were also carried out with the treatment of following reagents for 24 h: 10μM MAB1957 (integrin β3 blocking antibody, Millipore, USA), 5μM PF-573228 (FAK inhibitor, Selleck, USA). The control was SKOV3-NC and HO-8910-NC cell lines. Each migration and invasion assays was repeated three times on different days with different batches of cell (biological replicates).

### Wound healing assays

Cells were seeded in 6-well plates at a concentration of 1×10^6^ cells/well and grown overnight to confluent state. Then the monolayer was scratched using a sterile 200μl tip and cell debris were washed with PBS for three times. Microscope was used to detect the margin of the wound at 0 h and 24 h. Each cell line was assayed in biological triplicate.

### Cell adhesion assays

Exponentially growing cells were centrifuged, resuspended and seeded in 96-well plates coated with vitronectin (Sigma, Germany). After being incubated for 4 h, 8 h and 12 h at 37 °C, the non-adherent cells were removed using PBS. Adherent cells were fixed with CCK-8 reagent (10μl/well, Dojindo, Tokyo, Japan) and incubated for 3 h at 37 °C. The absorbance value of 450 nm was measured in Thermo Scientific Varioskan Flash (Thermo Fisher Scientific, USA). The curve was produced by averaging three experiments performed on different days using different batches of cells.

### Phospho-antibody microarray

The phosphor-protein array, using cell lysates of SKOV3-shCTHRC1 cells and SKOV3-NC cells as control, was performed by a Phospho-Antibody Array kit (CSP100, FullmoonBiosystems, CA) as previously described [27]. Briefly, cell lysates were biotin-labeled by biotin reagent in N, N-Dimethylformamide. Biotin-labeled samples were mixed with Coupling Solution. After incubation with blocking solution, the phospho-antibody array slides were conjugated to the protein coupling mix at 4 °C. The slides were washed with washing solution in triplicate, and incubated with Cy3-streptavidin solution at room temperature. The conjugation-biotin-labeled proteins were scanned using the GenePix 4000B (Axon Instruments, USA). The phosphorylation ratio was calculated as phosphorylation/unphosphorylation.

### Western blotting

To determine the protein expression, cells were lysed at 4 °C by RIPA supplemented with 1% phenylmethanesulfonyl fluoride, and 1% phosphatase inhibitor. Proteins were separated by 8–12% SDS-PAGE gel electrophoresis and transferred onto PVDF membranes. The membranes were blocked in 5% BSA for 1 h, and then incubated with primary antibodies at 4 °C overnight. Antibodies were CTHRC1 (Proteintech, USA), Integrin β3 (Abcam, UK), phospho-FAK (Tyr397) (Cell Signaling Technology, USA), FAK (Cell Signaling Technology, USA) and β-actin (Sigma, Germany) antibodies. Species-specific secondary antibodies were used to reveal the blots using Odyssey imaging system.

### Co-immunoprecipitation

In order to confirm the physical interaction between CTHRC1 and integrin β3, cell lysates pretreated using Protein A/G Sepharose beads (Sigma, Germany) were incubated with integrin β3 antibodies (Abcam, UK) and IgG as control at 4 °C overnight. The beads were washed by PBS containing 1% PMSF for three times, mixed with loading buffer at a ratio of 4:1 and incubated at 100 °C for 10 min. After centrifugation at 12,000 g for 3 min, the supernatants were tested by Western blotting.

### RNA extraction and real time RT-PCR assays

Total RNA was extracted with TRIzol reagent (Thermo Fisher Scientific, USA). Complementary DNA was synthesized using the random primers with a reverse transcription polymerase chain reaction kit (Applied Biosystems, CA) according to manufacturer’s instructions. Quantitative RT-PCR analyses were executed with specific primers using the SYBR Green PCR Master Mix (TaKaRa Biomedical Technology, Japan). The primers for CTHRC1 were 5′-TCATCGCACTTCTTCTGTGGA-3′ (forward) and 5′- GCCAACCCAGATAGCAACATC-3′ (reverse). The β-actin was detected as the internal control. The primers for β-actin were 5′-CCTGGCACCCAGCACAAT-3′ (forward) and 5′-GGGCCGGACTCGTCATAC-3′ (reverse).

### Immunohistochemistry

Immunohistochemical analysis (IHC) was carried out as described previously. Briefly, the 5μm sections were obtained from the paraffin-embedded human normal ovary tissues and ovarian cancer tissues of human or nude mice. The slides were incubated with CTHRC1 antibody (1:100, Proteintech), or integrin β3 antibody (1:400, Abcam) overnight, followed by incubation with HRP-labeled anti-rabbit antibody, and DAB for nucleus counterstaining with hematoxylin. Scoring of protein expression was measured by combining the percentage of positive cells (0, < 5% positive cancer cells; 1, 6–25% positive cancer cells; 2, 26–50% positive cancer cells; 3, 51–75% positive cancer cells; 4, 76–100% positive cancer cells) and intensity of staining (no staining scored 0; week staining scored 1; moderate staining scored 2; strong staining scored 3). The protein expression was defined by the final computation (the grades of extent × intensity of staining), low expression for the score of < 6 and high expression for the score of ≥6.

### Xenograft model

SKOV3 cells were stably transfected with Luc gene. For the in vivo metastasis assays, seven female BALB/c-nude mice (5-week-old) were injected i.p with 3 × 10^6^ SKOV3^luc^-Lenti-CTHRC1 cells, and five mice were injected with SKOV3^luc^-Lenti-NC cells as control. Each week, all mice were given 200μl D-luciferin to monitor the tumor progression using IVIS LuminaLT (Xenogen). Then, the mice were sacrificed and tumor tissues were preserved in −80 °C for further examinations, and dipped in neutral buffered formalin for the immunohistochemical study. Pooled tumors from multiple mice in each group were used for subsequent protein Western blotting and vitronectin-binding assays. For assays of anti-tumor metastasis effect of PF-228 FAK inhibitor, 14 female nude mice were divided into two groups (7 mice/group) and each group were injected i.p with 3 × 10^6^ SKOV3^luc^-Lenti-CTHRC1 cells. The mice in the treatment group were injected with PF-228 FAK inhibitor (50 mg/kg, i.p.) every other day. All animal experiments were carried out according to protocols approved by the Institutional Animal Care and Use Committee (IACUC) of Ren Ji Hospital, Shanghai Jiao Tong University School of Medicine.

### Statistical analysis

All statistical analyses were calculated by SPSS 16.0 software. All experiments were performed in triplicate. The data were presented as mean ± SD. The differences between two groups were analyzed by the double-sided Student’s t-test. The correlation between CTHRC1 and clinicopathological characteristics was assessed using the Chi-square test. *P*<0.05 was considered as statistically significant difference.

## Results

### CTHRC1 Enhances ovarian cancer cell migration and invasion in vitro

**Fig. 1:**
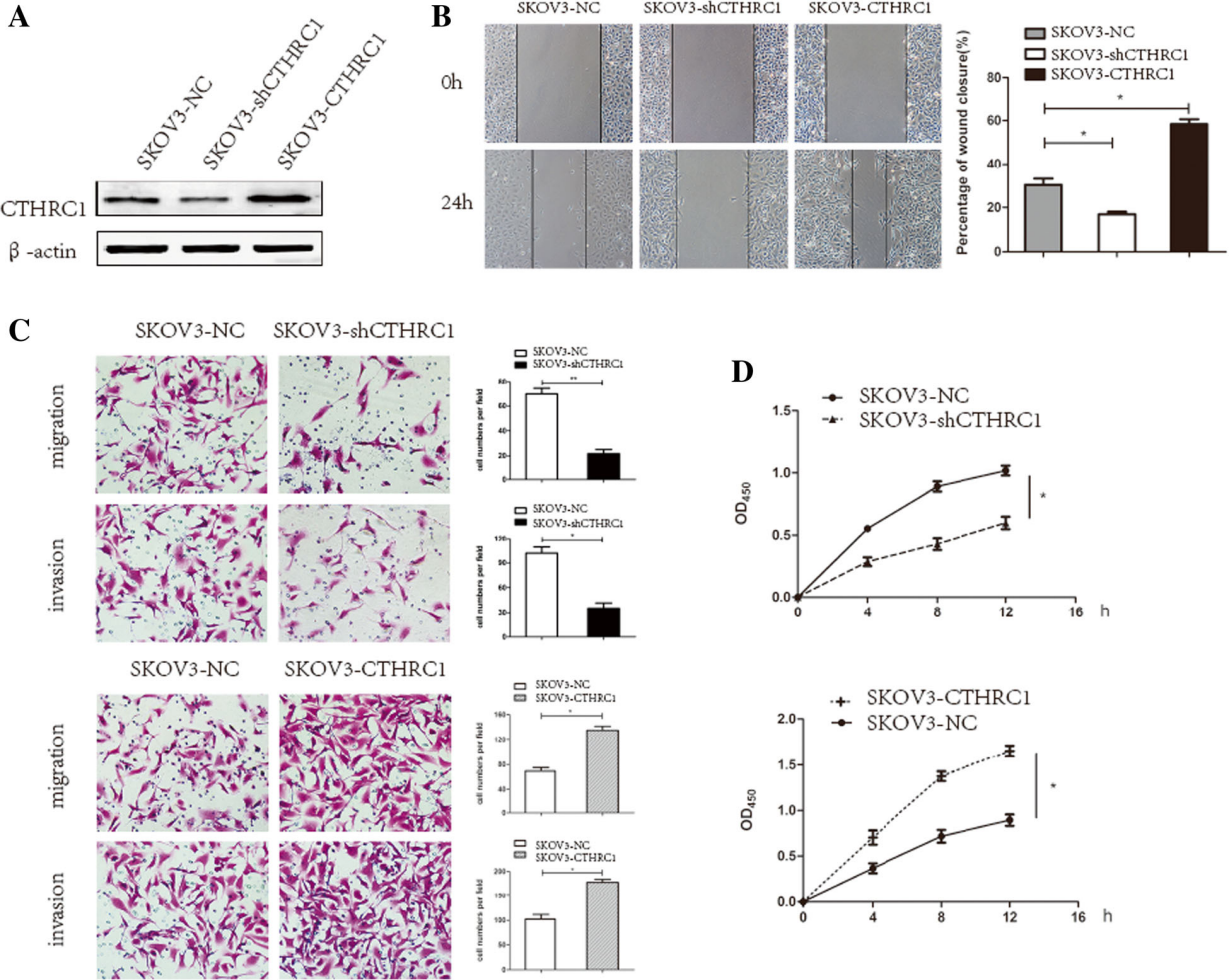
The effect of CTHRC1 on SKOV3 cells migration, invasion and adhesion in vitro. **a** The knockdown and overexpression of CTHRC1 in SKOV3 cells using CTHRC1-specific shRNA and Lenti-CTHRC1, respectively. **b** Decreased cellular migration in SKOV3-shCTHRC1 cells and elevated cellular migration in SKOV3-CTHRC1 cells were confirmed by wound healing assays. **c** Transwell migration and invasion assays showed that SKOV3 cells migratory and invasive capacity was impaired by down-regulation of CTHRC1 and enhanced by the up-regulation of CTHRC1. **d** Overexpressed CTHRC1 elevated SKOV3 cells adhesion to vitronectin and the knockdown of CTHRC1 reduced SKOV3 cells adhesion to vitronectin (*P < 0.05, **P < 0.01)

Previous study had pointed out that CTHRC1 expression is up-regulated in EOC patients [23]. To explore the effects of CTHRC1 expression in ovarian cancer cells, the expression was detected in immortalized ovarian superficial epithelium (IOSE) cells, and a panel of ovarian cancer cell lines. Compared to the IOSE cells, the expression of CTHRC1 was significantly up-regulated in SKOV3, A2780, ES2, and HO8910 cell lines (Additional file 1: Figure S1A). Furthermore, we established a set of human ovarian cancer cell lines in which CTHRC1 was stably up- or down- regulated. The lowest expression of CTHRC1 in ovarian cancer cell lines was observed in HO8910 cells, which was therefore stably transfected with Lenti-CTHRC1, thus obtaining the CTHRC1-overexpressing cell line, HO8910-CTHRC1. Furthermore, because of high expression of CTHRC1, SKOV3 cells appeared to be more suitable cell model for investigating metastasis in vivo [28], therefore SKOV3 cells was stably transfected with a CTHRC1-specific shRNA, thus generating the cell lines SKOV3-shCTHRC1. In addition, SKOV3 cells were stably transfected with Lenti-CTHRC1, thus obtaining the CTHRC1-overexpressing cell line, SKOV3-CTHRC1. Meanwhile, the control cell lines, HO8910-NC and SKOV3-NC, containing an empty vector were generated. We investigated the up- and down-regulation of CTHRC1 by Western blot (Fig. 1a and Additional file 1: Figure S1B).

The association between CTHRC1 and the cell migration and invasion were further investigated in vitro. Results from the wound healing assay demonstrated that after 24 h, the average area of clear zones for SKOV3-shCTHRC1 cells was larger than SKOV3-NC cells (Fig. 1b). SKOV3-NC cells had moved to fill 30% of the gap, while SKOV3-shCTHRC1 cells fill approximately 17% (*P* < 0.05). Moreover, the average area of clear zones for SKOV3-CTHRC1 cells and HO8910-CTHRC1 cells were smaller than the empty vector cells (*P* < 0.05, *P* < 0.01, respectively, Fig. 1b and Additional file 1: Figure S1C). In addition, compared with the corresponding empty vector cells (SKOV3-NC), the capacity of invasiveness and migration of SKOV3-shCTHRC1 cells were significantly decreased in both Boyden Chamber assays and Matrigel Transwell assays (Fig. 1c). Briefly, the invasive ability of CTHRC1 knocked out cells (SKOV3-shCTHRC1) was suppressed (*P* < 0.05) by 65%, and its migration capability was reduced (*P* < 0.01) by approximately 68%, compared with empty vector cells respectively. However, CTHRC1 over-expression in SKOV3 and HO8910 cells significantly enhanced cell invasion (*P* < 0.05, *P* < 0.01, respectively) and migration (*P* < 0.05, *P* < 0.01, respectively) capability (Fig. 1c and Additional file 1: Figure S1D). Additionally, our results suggested that the down-regulation of CTHRC1 expression had no effect on SKOV3 cells proliferation and colony formation in vitro (data not shown).

Since the interactions of tumor cells with the extracellular matrix (ECM) are a crucial step in invasion and metastasis, we further examined whether CTHRC1 expression could influence the adhesion capability of EOC cells. CTHRC1 over-expressed cells were seeded in the vitronectin (VTN)-coated 96-well plates. As shown in Fig. 1d, stable expression of CTHRC1 significantly enhanced (*P* < 0.05) SKOV3-CTHRC1 cell adhesion to vitronectin compared with empty vector cells (SKOV3-NC). Conversely, the number of adherent cells was obviously decreased (*P* < 0.05) in CTHRC1 down-regulation cells (SKOV3-shCTHRC1). Taken together, these results suggest that CTHRC1 is a positive metastatic regulator in EOC, and the over-expression of CTHRC1 could enhance the adhesion capability to cell-extracellular matrix.

### CTHRC1 Promotes EOC cells metastasis by activating integrin β3/FAK signaling

**Fig. 2:**
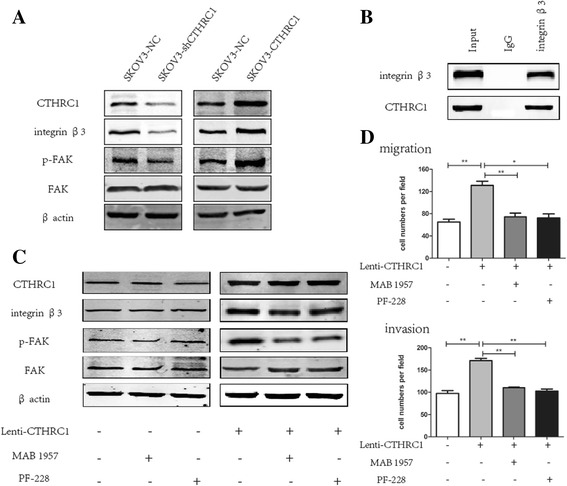
CTHRC1 activates the integrin β3/FAK signaling in SKOV3 cells. **a** The protein levels of integrin β3, p-FAK (Tyr397) and FAK in response to CTHRC1 knockdown and overexpression in SKOV3 cells were evaluated by Western blotting (β-actin was used for normalization). **b** Co-immunoprecipitation of integrin β3 and CTHRC1 in SKOV3 cells revealed that the endogenous CTHRC1 of SKOV3 cells was immunoprecipitated by integrin β3 antibody. **c** SKOV3-CTHRC1 and SKOV3 cells (control) were treated with MAB 1957 (anti-integrin β3 antibody) and PF-228 (inhibitor of FAK Tyr397 phosphorylation) for 24 h. PF-228 notably restrained the CTHRC1-induced phosphorylation of FAK (Tyr397); nevertheless, the level of integrin β3 protein wasn’t affected by MAB 1957. There was not any statistically significant changes of expression level of CTHRC1, integrin β3, p-FAK and FAK in SKOV3 cells after MAB 1957 and PF-228 treatment. **d** CTHRC1-induced cell migration and invasion was restrained by MAB 1957 and PF-228 (*P < 0.05, **P < 0.01)

In order to investigate the correlation between CTHRC1 and EOC metastasis, we performed a high-throughput phospho-proteome array to identify proteins whose phosphorylated forms were inhibited in SKOV3-shCTHRC1 cells (cells where CTHRC1 expression was down-regulated) compared with responding control cells SKOV3-NC. Briefly, the results from two independent experiments showed a spectrum of proteins whose phosphorylation levels were decreased more than 15% in SKOV3 cells when CTHRC1 was stably knocked down (Table 1). Many of these proteins, when phosphorylated, have been shown to be associated with cell migration, invasion, and tumor metastasis [4, 29–31]. Among these prometestatic proteins, the phosphorylation state of Focal adhesion kinase (FAK) on Tyr397 was dramatically decreased. Next, we confirmed by Weston blot that targeted down-regulation of CTHRC1 by shRNA resulted in reduced phosphorylation of FAK in SKOV3 cells (Fig. 2a). FAK, a nonreceptor tyrosine kinasen, and the integrin/FAK signaling pathway is an essential regulator of cell adhesion and migration. To characterize the signaling properties of CTHRC1 in EOC cell metastasis, we next focused on the phosphorylation level of FAK and the expression of upstream signaling molecules integrin β3 induced with CTHRC1 in SKOV3 cells. As shown in Fig. 2a, CTHRC1 over-expression increased the levels of integrin β3 and phosphorylated FAK, whereas knockdown of CTHRC1 expression decreased their levels. To further identify the relationship between CTHRC1 and integrin β3, we carried out co-immunoprecipitate analysis. Using the integrin β3 antibody, the endogenous CTHRC1 was apparently immunoprecipitated in SKOV3 cells (Fig. 2b).

**Table 1:** Proteins whose phosphorylation levels were decreased in SKOV3-shCTHRC1 cells compared with SKOV3-NC cells

Phosphorylation sites	Radio (SkOV3-shCTHRC1/SKOV3-NC)	95% CI
FAK(p-Tyr397)	0.62	0.60–0.64
STAT3(p-Ser727)	0.75	0.73–0.76
p38 MAPK (p-Tyr182)	0.76	0.70–0.82
Src(p-Tyr418)	0.78	0.75–0.81
Myc (p-Thr58)	0.79	0.67–0.92
STAT3(p-Tyr705)	0.80	0.76–0.86
HSP90B(p-Ser254)	0.81	0.78–0.83
c-Jun(p-Ser243)	0.84	0.79–0.89
4E–BP1 (p-Thr36)	0.85	0.80–0.91
NFkB-p65 (p-Thr254)	0.85	0.80–0.91

In the meantime, we verified the impact of integrin β3/FAK signaling upon the CTHRC1-induced migration and invasion of SKOV3 cells by using MAB1957 (anti-integrin β3 antibody) and PF-228 (inhibitor of FAK Tyr397 phosphorylation). Using MAB1957, which could specifically inhibit the function of integrin β3, the expression of integrin β3 was decreased slightly but with no statistical significance, while the phosphorylation of FAK (Tyr397) was significantly attenuated in SKOV3-CTHRC1 cells (*P* < 0.05, Fig. 2c). Analogously, the addition of PF-228 notably restrained the CTHRC1-induced phosphorylation of FAK (Tyr397); nevertheless, the level of integrin β3 protein wasn’t affected. Furthermore, we observed that the invasion and migration promoting effect of CTHRC1 was abolished by anti-integrin β3 antibody (MAB1957) and the inhibitor of FAK (PF-228) in SKOV3-CTHRC1 cells (Fig. 2d).

Above results suggested that CTHRC1 had physical interaction with integrin β3, and through enhancing the expression of integrin β3, CTHRC1 activated the phosphorylation of FAK at Tyr397. We made a further confirmation that CTHRC1 could promote ovarian cancer cells migration and invasion by activating the integrin β3/FAK signaling.

### CTHRC1 Promotes EOC cell intraperitoneal dissemination in vivo

**Fig. 3:**
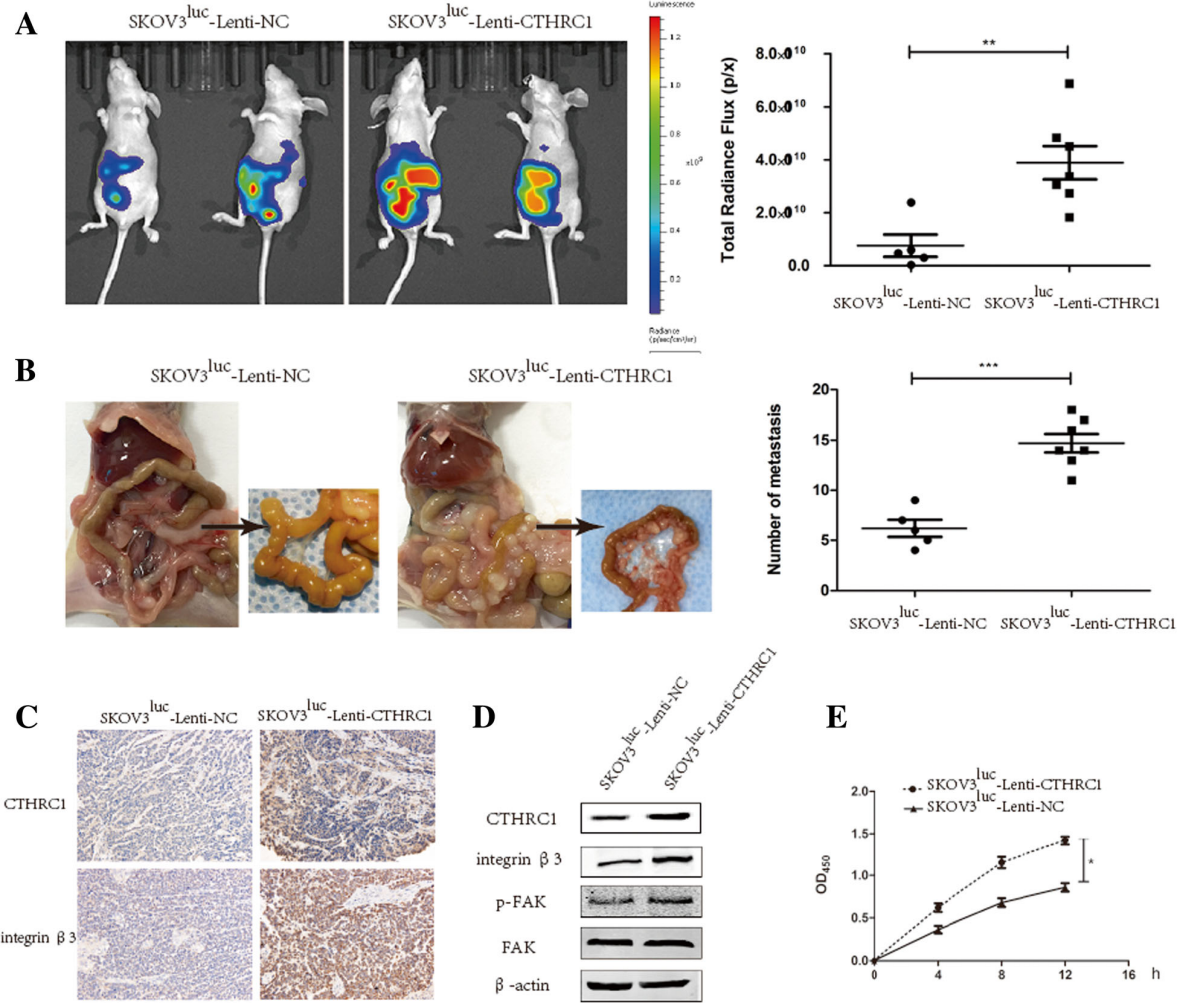
CTHRC1 promotes SKOV3 cells intraperitoneal dissemination in vivo. **a** and **b** Representative bioluminescent and anatomical images of intraperitoneal disseminated tumor in nude mice bearing SKOV3^luc^-Lenti-NC (*n* = 5) and SKOV3^luc^-Lenti-CTHRC1 (*n* = 7). Compared with mice injected with SKOV3^luc^-Lenti-NC, higher total radiance flux (**a**), and spread numerous metastatic tumors in the mesentery region, adjacent to the bowel and peritoneal wall (**b**), were observed in mice bearing SKOV3^luc^-Lenti-CTHRC1 cells (**P < 0.01, ***P < 0.001). Immunohistochemistry assay (**c**) confirmed that CTHRC1 overexpression in xenograft tumors (SKOV3^luc^-Lenti-CTHRC1 group) induced integrin β3 expression. Western blot assay (**d**) confirmed that CTHRC1 overexpression induced both integrin β3 expression and phosphorylation of FAK in xenograft tumors (SKOV3^luc^-Lenti-CTHRC1 group). **e** Cells pooled from multiple xenograft tumors in SKOV3^luc^-Lenti-CTHRC1 group brought out an increased adhesion to vitronectin compared with control group (*P < 0.05)

Here we examined whether stable over-expression of CTHRC1 increases EOC cell intraperitoneal dissemination in an in vivo ovarian cancer model*.* Based on our prior experience using i.p. xenograft models derived from SKOV3 cells i.p. injection [28], in this study disseminated ovarian cancer was generated by i.p. injecting female nude mice with human SKOV3^luc^-Lenti-CTHRC1 cells, while SKOV3^luc^-Lenti-NC cells were used as a control group. At 5 weeks later, we observed a significant difference in pattern of tumor development between two groups. A panel of representative images is shown in Fig. 3a-b. As Fig. 3a showed, the total radiance flux which reflected the orthotopic tumor and peritoneum metastasis was distinctly elevated (*P* < 0.01) in SKOV3^luc^-Lenti-CTHRC1 cells group (*n* = 7) compared with SKOV3^luc^-Lenti-NC cells group (*n* = 5). We also found that mice injected with SKOV3^luc^-Lenti-CTHRC1 cells spread numerous metastatic tumors to mesentery adjacent to the bowel and peritoneal wall, however, mice injected with SKOV3^luc^-Lenti-NC cells developed significantly few mesenteric implants (*15±2* (*n* = 7) vs. *6±2* (*n* = 5), *P* < 0.001, respectively, Fig. 3b). For the SKOV3^luc^-Lenti-CTHRC1 group, the ex vivo images confirmed the presence of the numerous tumor on the mesentery adjacent to the small bowel, while few tumor was detected in the control group. Moreover, the mice injected with control cells showed fewer incidence of metastasis in distant organ sites, whereas SKOV3^luc^-Lenti-CTHRC1-injected mice showed metastatic spread to spleen, liver, and stomach, excepting peritoneal wall. The pattern of tumor formation in the peritoneal space was consistent with the phenotype observed in vitro, suggesting an important role of CTHRC1 in promoting metastatic character of EOC cell.

In addition, we examined whether CTHRC1 interacts with integrin β3 in the mouse xenografts by Immunohistochemistry assays. As shown in Fig. 3c, CTHRC1 and integrin β3 were highly expressed in xenograft tumors of mice injected with SKOV3^luc^-Lenti-CTHRC1 cells. Western blot assays confirmed that CTHRC1 overexpression in xenograft tumors (SKOV3^luc^-Lenti-CTHRC1 group) induced both integrin β3 expression and phosphorylation of FAK (Fig. 3d). Cells derived from SKOV3^luc^-Lenti-CTHRC1 xenografts brought out an increased adhesion (*P* < 0.05) to vitronectin compared with controls (Fig. 3e). These results from in vivo suggested that over-expression of CTHRC1 leads to the up-regulation of integrin β3 in EOC xenograft tumor.

**Fig. 4:**
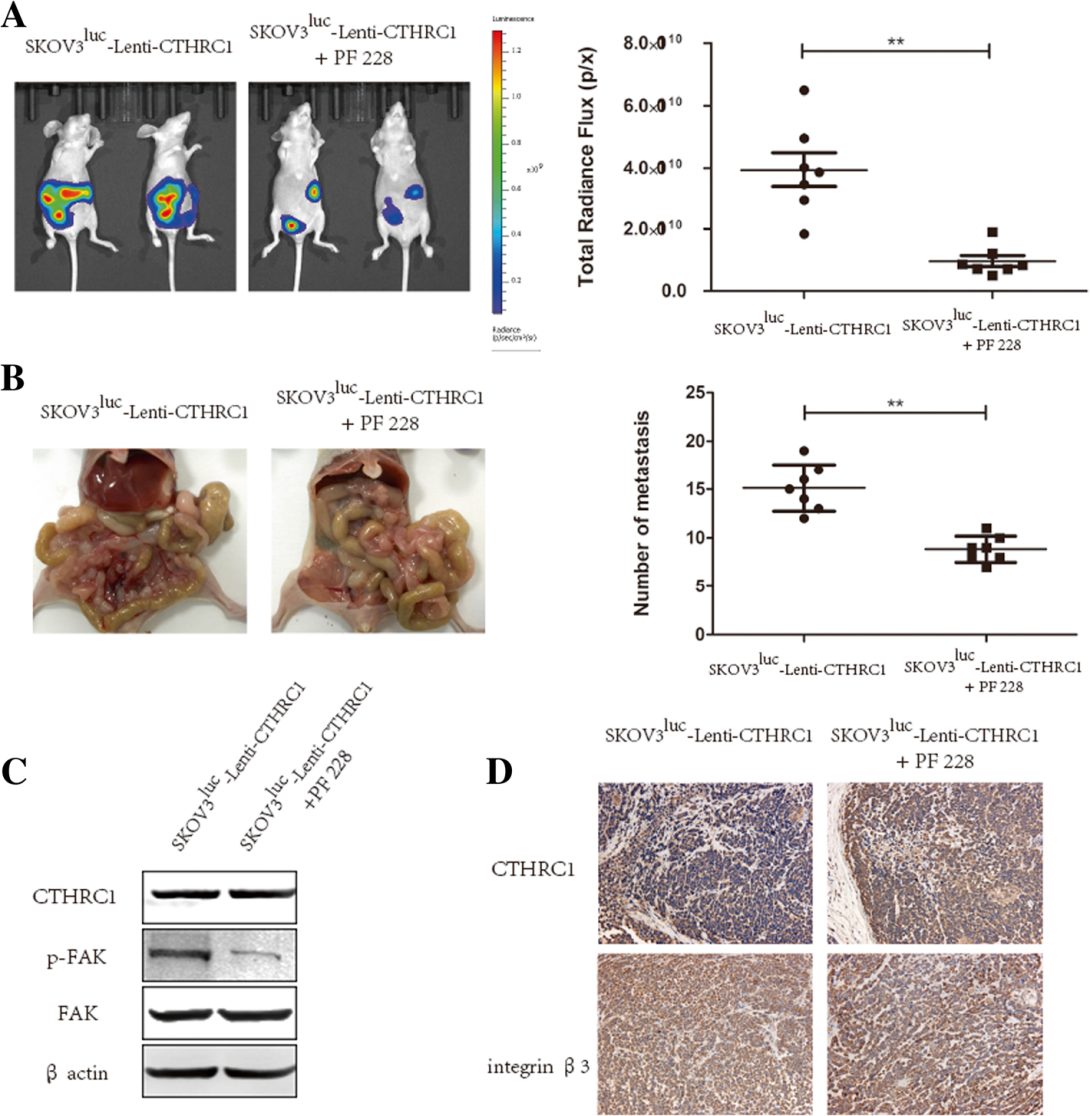
Inhibition of FAK phosphorylation attenuates SKOV3 cells metastasis in vivo. Mice bearing SKOV3^luc^-Lenti-CTHRC1 cells were i.p. injected with PF-228 for treatment group and vehicle as control, respectively. Total radiance flux (**a**) and metastases (**b**) were both declined in PF-228 treatment group. Using PF-228 significantly restored the impact of CTHRC1 on the phosphorylation of FAK (**c**), while integrin β3 protein levels had not been affected (**d**) in xenograft tumors (**P < 0.01, ***P < 0.001)

In converse experiments, we evaluated whether the stimulatory effects of CTHRC1 on EOC cell aggregation, implantation and migration in mouse model can be restored in the presence of PF-228 FAK inhibitor. We found that significantly reduced total radiance flux (*P* < 0.01), and number of abdominal metastases were observed in FAK inhibitor injected group, compared with the vehicle control group (9*± 1* vs. 15*± 2*, *P* < 0.001, Fig. 4a-b). In the meantime, we detected the expression of integrin β3 and the phosphorylation of FAK (Tyr397) in pooled tumors from multiple mice in each group. As shown in Fig. 4c-d, phosphorylated FAK was dramatically decreased in mouse xenograft tumors after PF-228 injection, while immunohistochemistry of mouse xenograft tumors showed that use of the FAK inhibitor had no impact on the expression of integrin β3 in vivo.

### CTHRC1 and integrin β3 signaling interaction in human EOC metastasis and clinicopathologic characteristics

**Fig. 5:**
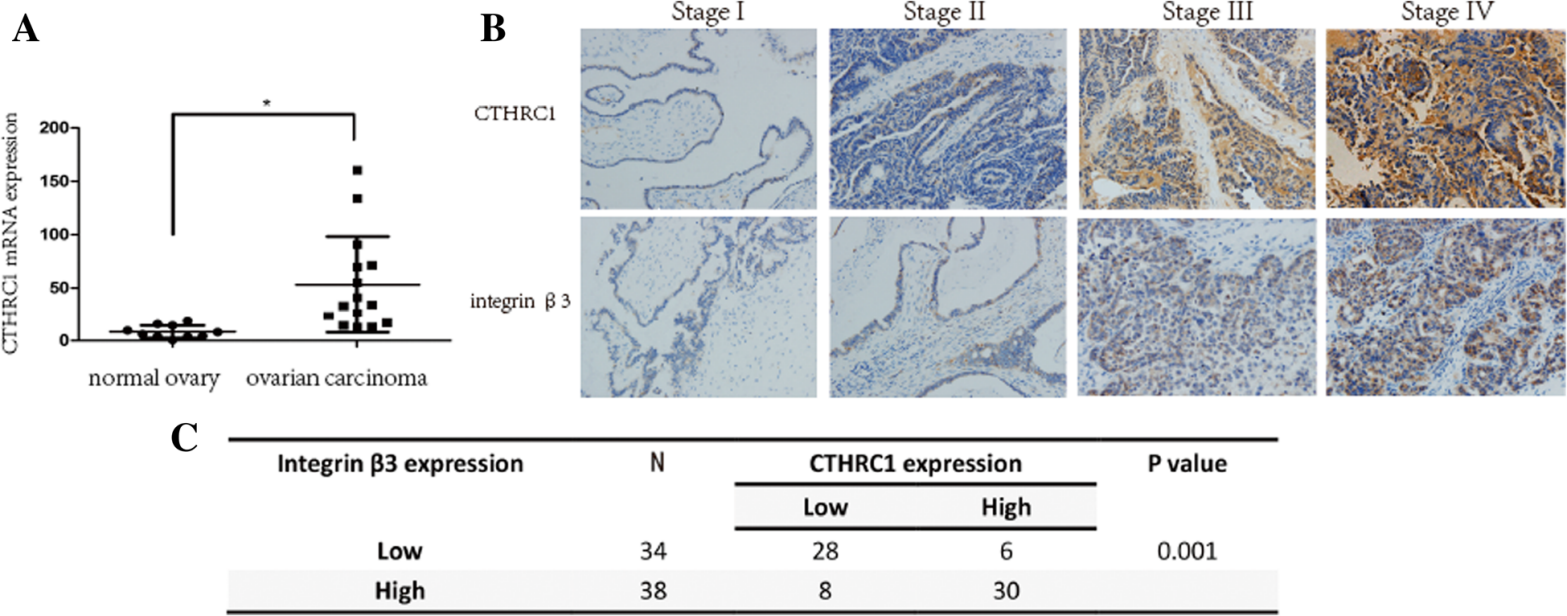
The expression of CTHRC1 and integrin β3 in human EOC tissues. **a** The mRNA levels of CTHRC1 were significantly higher in EOC tissue compared to normal ovary tissues (*P*< 0.05). **b** Representative IHC images for CTHRC1 and integrin β3 protein increased as the EOC progressed (FIGO I-IV). **c** Statistical analysis revealed a strong correlation between CTHRC1/integrin β3 co-expression (*P* = 0.001) and the progression and metastasis of EOC (*P < 0.05)

CTHRC1 is aberrantly over-expressed in multiple malignant tumors [20–23, 26]. To investigate the expression of CTHRC1 in human ovarian cancer tissue, we first examined the mRNA levels of Cthrc1 in 10 normal ovarian samples, and 15 epithelial ovarian cancer tissues using real time RT-PCR analysis. Compared to normal tissues, the expression level of CTHRC1 mRNA was significantly (*P*< 0.05) higher in EOC tissue than in normal tissue (Fig. 5a). The mRNA expression of CTHRC1 was very weakly detected in normal ovarian tissue, consistent with its expression in IOSE cells.

We further analyzed the protein expression and clinical significance of CTHRC1 in 72 ovarian cancer tissue samples obtained from patients by IHC. The results showed that CTHRC1 protein was increased as the disease progressed (FIGO I-IV) (Fig. 5b). As shown in Table 2, there was dramatical correlation between the CTHRC1 expression and FIGO clinical stage, lymph node metastasis, distance metastasis and ascites-derived cancer cells. However, the CTHRC1 expression wasn’t associated with patient’s age, tumor histological subtypes and tumor histologic grade. To define the predictive role of CTHRC1 expression in ovarian cancer metastasis, we performed Logistic regression analysis. The univariate analysis showed that CTHRC1 (*P* = 0.006), tumor grade (*P* = 0.111), histological subtypes (*P* = 0.068) and ascites-derived cancer cells (*P* = 0.018) might have influence on the metastasis, while the multivariate analysis confirmed that the CTHTRC1 expression (odds ratio (OR) = 3.66; *P* = 0.016) was an independent predictor of ovarian cancer metastasis (Tables 3 and 4). Simultaneously, the increasing expression of integrin β3 was observed with the progress of ovarian cancer too (Fig. 5b), and statistical analysis revealed a strong correlation between CTHRC1/integrin β3 co-expression (*P* = 0.001, Fig. 5c) and tumor metastasis. The over-expression of CTHRC1 in EOC tissues was strongly correlated with over-expression of integrin β3, suggesting that the increased expression of integrin β3 might result from up-regulation of CTHRC1 in human EOC. Both CTHRC1 and integrin β3 are good candidate markers for predicting progression and prognosis of ovarian cancer. Again, these results were consistent with the results from in vitro analysis confirming that CTHRC1 promotes EOC metastasis by activating integrin β3/FAK signaling.

**Table 2:** Correlation between the CTHRC1 expression and clinical characteristics in EOC

Clinical Characteristics	N	CTHRC1 expression		*P* value
		Low	High	
Age				0.083
< 50	25	9	16	
> 50	47	27	20	
Histological subtypes				0.336
Mucinous	5	4	1	
Serous	45	21	24	
Clear cell	4	1	3	
Endometrioid	16	8	8	
Others	2	2	0	
Tumor grade				0.239
High	18	7	11	
Medium	23	10	13	
Low	31	19	12	
FIGO stage				0.018
I-II	34	22	12	
III-IV	38	14	24	
Lymph node metastasis				0.001
Yes	24	5	19	
No	48	31	17	
Distance metastasis				0.001
Yes	34	10	24	
No	38	26	12	
Ascites-derived cancer cells				0.018
Yes	34	12	22	
No	38	24	14	

N, number of total samples in group

**Table 3:** Univariate Logistic regression analysis predicting metastasis of ovarian cancer in 72 patients

	B	OR	95%CI	*P*-value
age	0.541	1.718	0.646–4.572	0.278
Histological subtypes (serous +mucinous vs others)	0.965	2.625	0.931–7.402	0.068
Tumor grade (poor vs well/moderate)	0.772	2.163	0.837–5.595	0.111
Ascites-derived cancer cells	1.165	3.206	1.216–8.451	0.018
CTHRC1	1.392	4.021	1.505–10.741	0.006

**Table 4:** Multivariate Logistic regression analysis predicting metastasis of ovarian cancer in 72 patients

	B	OR	95%CI	P-value
CTHRC1 expression	1.298	3.661	1.272–10.534	0.016
Histological subtypes (serous +mucinous vs others)	0.999	2.716	0.872–8.456	0.085
Ascites tumor	0.806	2.240	0.785–6.387	0.132

## Discussion

Ovarian cancer is a significant cause of pelvic and peritoneal cavity metastasis, which is a devastating form of EOC progression with a dismal prognosis. There is no effective therapy for this condition, therefor it is crucial to identify novel prevention strategies, in addition to new markers necessary for understanding the molecular events involved in peritoneal metastasis status. CTHRC1 was initially identified in a screen for differentially expressed sequences in the balloon-injured adventitia and neointima versus normal arteries [19]. Secreted by fibroblasts and smooth muscle cells, CTHRC1 restrains the expression and deposition of collagen matrix, and enhances the cell migration [19, 32]. Recent studies have demonstrated that CTHRC1 is involved in cell adhesion and motility of various carcinomas [19, 33, 34]. In this research, we investigated the relationship between CTHRC1 and EOC metastasis in vitro and in vivo. Our results suggested that the knockdown of CTHRC1 suppresses the adhesion to vitronectin, migration and invasion of SKOV3 cells in vitro and vice versa. Meanwhile, the diffusion of SKOV3 cells in nude mice abdominal cavity was strengthened by the over-expression of CTHRC1 in i.p. xenograft model. More metastasis foci were found upon the mesentery adjacent to the bowel and peritoneal wall in the nude mice injected with SKOV3^luc^-Lenti-CTHRC1. Simultaneously, we revealed that the expression of CTHRC1 was associated with FIGO stage, lymph node metastasis, ascites-derived cancer cells and distance metastasis in EOC. In addition, univariate and multivariate logistic regression analysis suggested that CTHRC1 is an independent influential factor for ovarian cancer metastasis. Although the function of CTHRC1 in ovarian cancer cell metastasis is well-known, the mechanisms remained unclear. Hou et al. indicated that CTHRC1 activated the Wnt/β-catenin signaling to promote the EMT of epithelial ovarian cancer [23]. In this study, by using a microarray-based phosphor-antibody proteomics analysis, we distinguished a variety of proteins participating in tumor metastasis with phosphorylation that were down-regulated by the knockdown of CTHRC1. Among these pro-metastatic proteins, the inhibition of phosphorylation of FAK at Tyr-397 was the most remarkable. Chen et al. reported that CTHRC1 accelerated hepatic carcinoma cells adhesion and migration by up-regulating the expression of integrin β1, and the phosphorylation of FAK [21]. Park et al. demonstrated that CTHRC1 promoted the Src-FAK complex formation and the activation of FAK in pancreatic cancer [25]. Conformably, our research implicated that CTHRC1 can mediate the activation of FAK. Previous studies have proved that integrins are the prime regulators of FAK [14, 35]. Through binding to arginine-glycine-aspartic acid (RGD) containing molecule of ECM and recruiting downstream targets, integrin/FAk signaling promotes ovarian cancer cells attachment and metastasis [36, 37]. In this study, we verified that the knockdown of CTHRC1 inhibits the expression of integrin β3, and the phosphorylation of FAK. In contrast, ectopic expression of CTHRC1 leads to the activation of integrin β3/FAK signaling in vitro and in vivo. Moreover, we demonstrated that CTHRC1 interacts with integrin β3 physically, which furthermore attests the mechanism of CTHRC1 in ovarian cancer cell. Also, our IHC assessment of ovarian cancer from patients and xenograft tumor tissues showed a strong correlation between the co-expression of CTHRC1 and integrin β3 as the tumor progression.

**Fig. 6:**
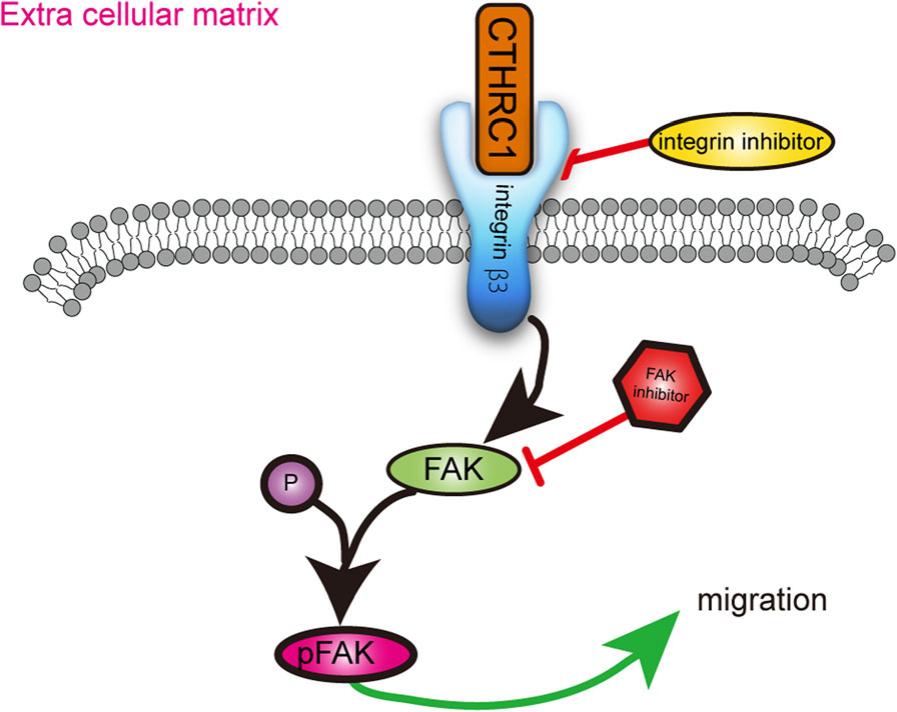
Extra cellular matrix

Previous researches have demonstrated that targeting the integrin β3 /FAK signaling could enhance the anti-tumor activity, and attenuate cancer metastasis including melanoma, endometrial cancer, non-small-cell lung cancer and esophageal squamous cell carcinoma [18, 38–42]. Consequently, integrin β3 blocking antibody MAB1957 and the FAK inhibitor PF-228 were used to verify whether the inhibition of integrin β3 function and FAK phosphorylation would restore the influence of CTHRC1 on ovarian cancer cell migration and invasion. We observed that both MAB1957 and PF-228 could attenuate the phosphorylation of FAK at Tyr-397 and dramatically decline the capability of migration and invasion in CTHRC1 overexpressed cells in vitro (Fig. 6). Our results were identical with previously referenced study where the inhibition of integrin β3 and FAK decreased the migration and invasion of other solid tumor. Furthermore, we investigated the function of FAK inhibitor PF-228 in ovarian cancer metastasis in vivo*.* The nude mice injected with SKOV3^luc^-Lenti-CTHRC1 cells developed less peritoneal metastases after using PF-228, which further confirmed that CTHRC1 induced cancer metastasis through activating the phosphorylation of FAK.

## Conclusion

To sum up, our results provide first evidence that CTHRC1 interacts with integrin β3 and accelerates the FAK phosphorylation to promote ovarian cancer cell adhesion, migration and invasion in vitro and in vivo*.* The correlation between CTHRC1 and integrin β3/FAK signaling exposes the mechanisms underlying peritoneal ovarian tumor dissemination, and provides a new direction in ovarian cancer diagnosis and treatment.

## Additional file

Additional file 1: Figure S1. The expression and effect of CTHRC1 on EOC cells migration and invasion in vitro. (A) Compared to IOSE cells, the protein levels of CTHRC1 in ES2, SKOV3, A2780 and HO8910 cell lines were significantly up-regulated. (B) The overexpression of CTHRC1 in HO8910 cells using Lenti-CTHRC1. (C) Wound healing assay showed an increased cellular migration in HO8910-CTHRC1 cells. (D) Elevated cellular migration in HO8910-CTHRC1 cells were confirmed by Transwell migration and invasion assays. (***P* < 0.01). (TIFF 959 kb)

## Abbreviations

CTHRC1: Collagen triple helix repeat containing 1
CXCLs: CXC chemokine ligands
CXCRs: Chemokine receptors
ECM: Cell-excretal cellular matrix
EMT: Epithelial-mesenchymal transition
EOC: Epithelial ovarian cancer
ERK: Extracellular signal-regulated kinase
FAK: Focal adhesion kinase
FBS: Fetal bovine serum
HCC: Hepatocellular carcinoma
i.p.: Intraperitoneal injection
IOSE: Immortalized ovarian surface superficial epithelium
MMP9: Matrix metalloproteinase 9
MMPs: Matrix metalloproteinases
PDAC: Urokinase-type plasminogen a pancreatic ductal adenocarcinomas
PEOC: Primary epithelial ovarian cancer
Src: Steroid receptor coactivator
uPA: Urokinase-type plasminogen activator
