# Supplementary figures and images for: Collagen triple helix repeat containing 1 (CTHRC1) activates Integrin β3/FAK signaling and promotes metastasis in ovarian cancer
Source: J Ovarian Res. 2017 Oct 11;10:69. doi: 10.1186/s13048-017-0358-8 (PMC5637322; doi:10.1186/s13048-017-0358-8)

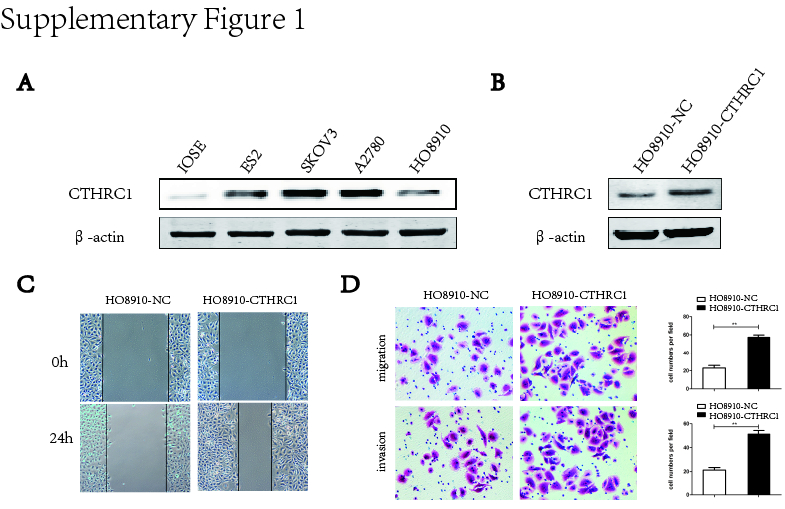

Supplement: Additional file 1: Figure S1. — The expression and effect of CTHRC1 on EOC cells migration and invasion in vitro. (A) Compared to IOSE cells, the protein levels of CTHRC1 in ES2, SKOV3, A2780 and HO8910 cell lines were significantly up-regulated. (B) The overexpression of CTHRC1 in HO8910 cells using Lenti-CTHRC1. (C) Wound healing assay showed an increased cellular migration in HO8910-CTHRC1 cells. (D) Elevated cellular migration in HO8910-CTHRC1 cells were confirmed by Transwell migration and invasion assays. (**P < 0.01). (TIFF 959 kb) [file 13048_2017_358_MOESM1_ESM.tif]
